# The Effectiveness of Currently Recommended Questionnaires in Identifying Scoliosis Among Chronic Back Pain Patients: A Cross-Sectional Study

**DOI:** 10.3390/healthcare13243196

**Published:** 2025-12-05

**Authors:** Fabio Zaina, Tito Bassani, René Castelein, Carmelo Pulici, Stefano Negrini

**Affiliations:** 1 ISICO (Italian Scientific Spine Institute), 20141 Milan, Italy; carmelo.pulici@isico.it; 2 IRCCS Galeazzi—Sant’Ambrogio Hospital, 20157 Milan, Italy; tito.bassani@grupposandonato.it (T.B.); stefano.negrini@unimi.it (S.N.); 3 Department of Orthopedic Surgery, University Medical Center Utrecht, 3508 GA Utrecht, The Netherlands; r.m.castelein-3@umcutrecht.nl; 4 Department of Biomedical, Surgical and Dental Sciences, University “La Statale”, 20149 Milan, Italy

**Keywords:** adult scoliosis, chronic low back pain, quality of life

## Abstract

Background/Objectives: Low back pain (LBP) is the most prevalent musculoskeletal condition, significantly impacting quality of life and incurring high social costs. Although non-specific (without anatomical abnormalities) LBP accounts for nearly 80% of cases, LBP due to adult spinal deformities (ASDs), including scoliosis, remains a major concern. Several patient-reported outcome measures (PROMs)—notably the Oswestry Disability Index (ODI), Scoliosis Research Society-22 questionnaire (SRS-22), and Core Outcome Measure Instrument (COMI)—are recommended for assessment in these populations. This study aims to verify if these PROMs can effectively distinguish between adults with scoliosis-associated LBP (SLBP) and those with non-specific LBP (LBP). Methods: subjects were categorised as either having idiopathic/degenerative scoliosis (>10° Cobb angle in the coronal plane) with LBP, or non-specific LBP. Statistical comparisons applied non-parametric tests (Wilcoxon rank-sum, Mood’s median, chi-square), Spearman’s correlation, and generalised linear regression analyses. Results: Among 1092 subjects (552 SLBP; 540 LBP), median ODI scores were similar between groups, while SRS-22 scores were modestly higher in the SLBP cohort. Females consistently reported higher ODI and lower SRS-22 scores. Significant correlations arose between ODI and COMI, with moderate inverse associations with SRS-22. Regression analysis demonstrated that pathology group, gender, age, and BMI weakly predicted PROM scores. Conclusions: ODI and SRS-22 perform comparably in assessing disability in adults with LBP regardless of scoliosis, suggesting they cannot discriminate different pathologies. These findings underscore the importance of employing multiple PROMs to capture clinical dimensions.

## 1. Introduction

Low back pain (LBP) is the most common musculoskeletal condition with a relevant impact on quality of life and social costs [1]. The World Health Organisation (WHO) has included LBP among the chronic diseases needing a global rehabilitation approach by 2030 [2]. Most cases of LBP have no clear and proven cause and are defined as non-specific LBP [3]. This condition accounts for approximately 80% of cases. For the remaining part, a specific cause can be found. Beyond non-specific cases, structural alterations of the spine account for a substantial proportion of low back pain, and Adult Spinal Deformities (ASDs) are probably the main cause of specific low back pain [4]. They are defined as a group of alterations of the spinal alignment, including adult scoliosis, degenerative scoliosis, sagittal and coronal imbalance, and iatrogenic deformity, with or without spinal stenosis [5]. The prevalence of these conditions increases with age. For scoliosis, it goes from about 3% [6] of adolescents to 32% in adults and up to more than 68% in the elderly [7,8].

Low Back Pain is defined as both a syndrome and a symptom [9]. So, it is clear that a person with ASD can suffer from both non-specific LBP and pain caused by its pathology. It is not always easy to distinguish between them. According to a recent systematic review, the pain pattern connected to scoliosis is more asymmetric, eventually radiating to one thigh. At the same time, non-specific LBP is usually more symmetric, eventually radiating to one leg and foot [10]. Moreover, standing and walking are some of the most compromised activities in patients with low back pain associated with scoliosis [10].

Over time, specific tools have been developed to assess the extent of the impact of these conditions. The Oswestry Disability Index (ODI) was developed to assess the quality of life in adult patients with non-specific chronic LBP. The ODI explores various aspects of daily life, from standing to walking, driving, and personal care. Additionally, some studies suggest that the ODI can better capture the condition of patients with spinal pain and deformities than other, more specific tools [11]. The SRS-22 questionnaire is the most commonly used instrument to evaluate the quality of life (QoL) in patients with idiopathic scoliosis [12]. It has been translated into several languages and has proven valid, especially among adolescents. For adult patients, the SRS-22 questionnaire remains the most utilised instrument for QoL assessment, although fewer studies have examined it within this population. Both SRS-22 and ODI are among the PROMS recommended for adult patients with spinal deformities and low back pain [13]. More recently, a further tool called COMI (Core Outcome Measure Instrument) was developed, originally for low back pain but also applied in ASD [14]. Comparisons between various instruments are scarce in the literature [15], and the advantages of different questionnaires are not known with absolute certainty. Specifically, it is not entirely clear how the ODI performs in cases of spinal deformity and whether there are differences concerning the SRS-22 in subjects with non-specific low back pain or secondary to scoliosis. The same is true for the COMI.

Our Institute’s clinical protocols involve the regular and continuous assessment of the quality of life of patients undergoing treatment and monitoring for LBP, scoliosis and other spinal deformities. For this purpose, the SRS-22, ODI, and COMI questionnaires, as well as other measurement tools such as the Italian Spine Youth Quality of Life (ISYQoL), have been progressively implemented and administered to our patients. The latter was initially used for underage patients, but since some of them continue therapy and monitoring beyond the age of 18, we have continued its use in these subjects [15].

This study aims to compare the properties of the ODI and the SRS-22 questionnaire in adults by comparing subjects with non-specific low back pain and those with LBP and scoliosis. We want to compare the performance of these questionnaires in patients with and without scoliosis. The secondary aim of this study is to conduct a similar assessment for COMI. Finally, we aim to compare the properties of the ODI, the SRS-22, and the COMI questionnaire in adults with scoliosis and chronic low back pain, based on the severity of the curve, dividing curves into larger (30° Cobb and above) and smaller (below 30°) categories.

## 2. Materials and Methods

### 2.1. Study Design

This is a cross-sectional observational cohort study.

### 2.2. Population

Consecutive adult patients who came to our Institute from 2017 to the date of approval of this protocol, who have completed the SRS-22 and the ODI questionnaires, and who met the following inclusion criteria:

- Age ≥ 50 years
- Diagnosis of idiopathic or degenerative scoliosis with a curve >10° Cobb and low back pain or
- Diagnosis of non-specific low back pain

The exclusion criteria were:

- Previous spinal surgery
- Previous and significant illnesses, surgeries, or traumas
- Secondary scoliosis
- Other deformities, such as spondylolisthesis and symptomatic canal stenosis (diagnoses clinically and radiographically)
- Incomplete ODI and SRS-22 data

All patients signed a written consent form to participate in the study, which was approved by the local Ethics Committee (Comitato Etico Territoriale Lombardia 3, parere 4762_18.12.2024_P_bis) and registered on Clinicaltrials.gov (NCT06779240).

### 2.3. Sample Size

The sample size estimation was performed using a priori power analysis with G*Power software (version 3.1.9.7, Düsseldorf, Germany). Because the literature does not provide comparable values for expected differences, average reference values for effect size parameters were used. For the primary objective—identifying a significant difference in the mean total score of the SRS-22 and ODI questionnaires between patients with non-specific low back pain and those with idiopathic or degenerative scoliosis—the sample size was evaluated using a two-tailed Wilcoxon–Mann–Whitney non-parametric test. This test was chosen because it is more restrictive than the *t*-test for independent samples. A medium effect size (d = 0.5), significance level (α = 0.05), statistical power (0.8), and balanced groups were assumed. The estimate indicated a required sample of 67 subjects per group.

For the analysis of the Pearson correlation coefficient (r), the number of subjects was adequate to detect a statistically significant value of r > 0.3 using an exact one-tailed test with α = 0.05 and power = 0.8. The same analysis was applied for the evaluation of the secondary objective (difference in mean values between subjects with curve <30° and >30° in the group with scoliosis), resulting in a total number of 134 subjects (672).

This sample size is also adequate for the multiple linear regression model to assess the association between predictive variables and questionnaire scores. In this case, a two-tailed *t*-test approach was used to verify the statistical significance of the single coefficient with the following parameters: fixed model, effect size f^2 = 0.15, α = 0.05, power = 0.8, up to 20 predictive variables.

To allow for multiple comparisons, we included all subjects with complete data who met the inclusion criteria.

### 2.4. Statistical Analysis

As the data distributions were generally non-normal, we conducted comparisons of median values between pathology groups and within each pathology group by gender. We tested for median differences using the Wilcoxon rank-sum test to determine statistical significance. We tested differences in gender proportions between groups using the chi-square test. We analysed median values for ODI and SRS-22 total scores with the Wilcoxon rank-sum test, while specific item scores and COMI Back scores were analysed using Mood’s median test. The latter approach was chosen because specific item responses are limited to a narrow range of ordinal values, making the Wilcoxon test unsuitable. Mood’s test evaluates differences based on chi-square testing of the proportion of values exceeding the overall median.

The relationships among ODI, SRS-22, and COMI Back total scores were assessed using Spearman’s correlation coefficients. The statistical significance of these coefficients was tested using permutation distribution tests. Differences in coefficients between pathology groups or genders within a group were analysed using Fisher’s z-test.

The predictive effects of pathology group, gender, age, and BMI on ODI and SRS-22 total scores were evaluated using generalised linear regression models. Group and gender were treated as categorical variables. The contribution of each variable was quantified through estimated coefficients and tested for statistical significance using *t*-tests. Partial correlations between independent variables and ODI or SRS-22 total scores were calculated using Spearman’s correlation coefficients.

All analyses were performed using MATLAB software (v.R2024b, MathWorks Inc., Natick, MA, USA).

## 3. Results

The dataset included 1092 subjects divided into two pathology groups: scoliosis with low back pain (SLBP) and non-specific low back pain (LBP).

### 3.1. Comparison Between the Two Pathology Groups, SLBP and LBP

The dataset included 552 subjects in the SLBP group and 540 in the LBP group (Table 1). Females were more prevalent in the SLBP group than in the LBP group. The age and BMI were similar between the groups. Among genders, females had slightly lower BMI than males in both groups and exhibited higher Cobb angles in the SLBP group (median 41° for females vs. 29° for males).

The pathology groups‘ ODI total scores and specific item scores were similar (Table 2). Median ODI total scores were 24 (IQR 22) in the SLBP group and 24 (20) in the LBP group. Females had higher total scores than males in both groups: 26 (22) vs. 17 (19) in SLBP, and 26 (20) vs. 22 (18) in LBP (Appendix A—Table A1). Specific item scores showed higher values in females compared to males for pain, lifting, walking, and employment/homemaking in both the pathology groups.

SRS-22 data were available for 469 subjects (357 SLBP, 112 LBP; Table 3). This smaller number is due to a lack of standardisation in the administration of the questionnaires at our Institute in the past. We reduced the risk of biased selection by comparing the data of those who completed and those who did not complete the SRS-22 (Appendix A—Table A2). For the SRS-22, the total scores were slightly higher in the SLBP group: 60 (22) vs. 56 (21) in the LBP group. However, specific item medians were similar due to imbalanced distributions. Females showed lower total scores in both groups: 60 (22) vs. 66 (18) in SLBP, and 55 (21) vs. 66 (22) in LBP (Appendix A—Table A3).

COMI Back total scores and specific item scores were similar between pathology groups (Table 4). However, females had higher median scores than males in both groups.

ODI and COMI scores were strongly positively correlated, while both showed moderate negative correlations with SRS-22 scores (Table 5). Correlation values were slightly larger for ODI and SRS-22 in the pathology groups (−0.63 and −0.52, respectively), and very similar for ODI and COMI (0.77 and 0.8) and SRS-22 and COMI (−0.6). Gender-specific correlations showed minimal differences.

Pathology group, gender, age, and BMI had statistically significant but weak predictive effects on ODI and SRS-22 total scores (Table 6). LBP was weakly associated with higher ODI scores and lower SRS-22 scores compared to SLBP. Females had higher ODI scores and lower SRS-22 scores than males. Increased age and BMI were associated with higher ODI scores and lower SRS-22 scores. Partial correlations of these variables with ODI and SRS-22 total scores were low (absolute values ranging from 0.06 to 0.21). Results were consistent across subgroup analyses (Table 7).

### 3.2. Comparison Between the Two Scoliosis Severity Subgroups Within the SLBP Group

A higher prevalence of females was observed in the subgroup with a Cobb angle greater than 30° (Table A4—Appendix A). The ODI total score was higher in the Cobb angle >30° group compared to the <30° group, with median (interquartile range) values of 24 (18) and 20 (22), respectively. Females exhibited higher ODI scores than males across both scoliosis severity groups.

SRS-22 data were available for 268 subjects (75 with a Cobb angle <30° and 193 with a Cobb angle >30°). Total scores were marginally higher in the <30° group: 64 (29) versus 61 (19), although this difference was not statistically significant. Specific item medians were generally comparable between groups, except for self-image, which showed a decrease with increasing scoliosis severity (15 vs. 10). Females had lower total SRS-22 scores than males in both severity groups: 63 (28) vs. 69 (32) in the <30° group and 61 (20) vs. 66 (13) in the >30° group.

COMI Back scores were similar across scoliosis severity subgroups but tended to be slightly higher in females compared to males.

Correlation analysis demonstrated relationships among the ODI, SRS-22, and COMI Back total scores comparable to those previously reported in the SLBP group. These values were similar across subgroups of scoliosis severity and gender.

Predictive analysis confirmed that increased scoliosis severity was associated with higher ODI scores, but this association was not statistically significant with the SRS-22 score. However, the correlation coefficient was generally low (r = 0.11). Females provided larger ODI scores in the <30° group, as well as larger age and BMI variables. The independent variables also provided lower SRS-22 but only in the >30° group (Appendix A—Table A5), with similar correlation values (ranging from 0.12 to 0.25 in absolute value).

## 4. Discussion

PROMS are recommended and necessary for a complete evaluation of patients with spinal issues. In the past, a combination of general health evaluations and more specific scales and tools was recommended, including the VAS and ODI [16]. The Scoliosis Research Society, based on these original indications, developed the “Scoliosis Research Society adult spinal deformity standard outcome set”, which includes the ODI, the SRS-22 and an NRS for leg and pain, together with a more general health questionnaire like the EQ-5D 3L [13]. More recently, new tools like the PROMIS have been developed; however, a consensus on their application is still lacking, and their cultural adaptation is not available in many countries [17]. In our Institute, we routinely use the ODI, SRS-22, and the COMI, which is designed to assess pain, function, and quality of life in patients with spinal disorders. It includes two GRS for leg and back pain. This approach is consistent with the current recommendations, and since most of the research on these tools has been produced in a surgical setting, we aimed to report data in a rehabilitation setting. We compared the ability of the ODI and SRS-22 questionnaires to discriminate scoliosis and non-scoliosis in adult patients with chronic Low Back Pain. Our results showed similar performance in both pathologies, meaning they cannot discriminate between them.

Our results indicate that ODI total scores were comparable between the SLBP and LBP groups. Based on this tool, we can say that the level of disability reported by patients with scoliosis is similar to that of those with non-specific low back pain. To our knowledge, this is the first comparison between these populations made with the ODI. We hypothesise that the response could have been different and that the scoliosis patients would have shown lower scores in some specific items, but this was not the case. The ODI has already been used in studies comparing patients with adult scoliosis to healthy controls [18,19]. The findings showed that healthy controls performed better; however, the sample was significantly younger than our population.

The SRS-22 total score was slightly higher in the SLBP group. This could potentially reflect an influence of self-image and mental health components that are more relevant in scoliosis populations. Females with larger scoliosis showed the lowest scores compared to all other groups; however, the difference did not reach statistical significance. These findings raise different points. It appears that patients with scoliosis and larger curves may present a specific burden that appropriate questions could address. The point is not simply about the diagnosis of scoliosis, but the entity of the curve can relate to the physical appearance. Patients with curves larger than 30° had lower self-image scores. This seems consistent with a recent paper that found a negative correlation of the main thoracic Cobb angle and several other clinical parameters with self-image and mental health [20]. On the other hand, the previous literature is inconsistent regarding the correlation between the Cobb angle and self-image [21]. An objective evaluation of the trunk appearance in these patients would likely aid in a deeper understanding of this point.

Gender differences were observed in both groups, with females reporting significantly higher ODI scores and lower SRS-22 scores than males. These findings are consistent with other studies reporting higher scores on the SRS-22 in males with scoliosis compared to females [22]. Pathology group, gender, age, and BMI significantly but weakly predicted ODI and SRS-22 scores. This means that some variety exists within the broader groups of SLBP and LBP. There are reports on the differences between male and female perception and description of pain. When referring to pain, women tend to employ more detailed and factual language, indicative of heightened emotional sensitivity. In contrast, men tend to use fewer words and focus on the sensory aspects of pain [23]. According to a review, gender differences may exist in pain tolerance, pain sensitivity, pain threshold, and responsiveness to pain [24]. This is consistent with other reports showing that males and females respond differently to the ODI and other evaluation tools [25]. Additionally, age is a factor that should be considered. Our data demonstrates small differences, but it is worth exploring further. Probably the differences are multifactorial.

Strong positive correlations were found between ODI and COMI Back scores, while both were moderately negatively correlated with SRS-22. These results suggest that ODI and COMI capture overlapping aspects of disability and pain perception, whereas the SRS-22 provides distinct information related to scoliosis-specific quality of life. It is quite surprising that the SRS-22, a highly specific tool, performed similarly in ASD and non-specific LBP. Doubt arises about the ability of such a tool to precisely monitor all the characteristic issues of ASD. Generally speaking, there is a global effort to try to find better ways to assess the function and QoL of patients with spinal problems. This is documented by the PROMIS project [17], the development of the COMI [26], and other questionnaires, such as “The forgotten spine score” [27]. Our findings are consistent with this perspective, and question whether we are focusing on the real issues of ASD patients and whether we are using the right tools.

In recent years, the focus of research has started looking at the role of the sagittal balance in ASD as a predictor of disability and pain [8]. Earlier studies found the pelvic parameters to be more relevant than the coronal Cobb angle in predicting the negative clinical impact of scoliosis [28]. Moreover, recent evidence suggests that the Spino Sacral Angle (SSA) also has a predictive role for disability measures, as indicated by the ODI [29]. ASD refers to a wide range of patients with diverse features [30]. We can speculate that all these elements, such as age, sex, BMI, curve size, localisation, and sagittal balance, contribute to the clinical limitations of patients, with none predominant over the others. Unfortunately, we were unable to retrieve the lateral radiographs of many of these patients, and therefore, we cannot confirm this hypothesis with our data. Nevertheless, this is beyond the scope of this paper.

From a clinical perspective, these findings also emphasise the importance of using multiple PROMs to understand a patient’s condition comprehensively. Theoretically, while ODI is effective for assessing functional disability, it may not fully capture the psychosocial impact of scoliosis. The SRS-22 provides valuable information in this regard but may not be as relevant for patients with non-specific low back pain. Our results suggest that combining these instruments with a measure like COMI can enhance the evaluation of the physical and psychological dimensions of spinal disorders.

For clinical research, the point is consistent. Due to the specific features and the limitations of each tool, using multiple questionnaires seems the most appropriate way to capture all the different peculiarities and the impact of spinal pathologies. Such an approach is consistent with previous suggestions in the field of spinal deformities [13,16] and LBP [31].

This study has some limitations. The main one is the retrospective analysis of data, which may introduce selection bias. We could have also missed other relevant information that could have created a more specific subgroup with different responsiveness. Nevertheless, because the aim is to evaluate the performance of different questionnaires to discriminate among different populations, this approach seems adequate to increase the generalizability of our findings. Additionally, longitudinal studies are needed to assess how these PROMs perform over time and in response to treatment interventions. Not all patients completed the SRS-22. This is due to the retrospective design and the freedom given to patients to complete or not complete the questionnaires. This may introduce a selection bias, but we ruled it out by comparing the data of those who filled it and those who did not. Another limitation is the absence of sagittal balance parameters, which are known to influence functional outcomes. This omission restricts interpretation regarding the biomechanical contribution to reported disability.

Further research should investigate additional factors that influence patient-reported outcomes, including pain duration, psychological distress, and treatment history. Incorporating qualitative assessments may provide deeper insights into patients’ experiences and inform more patient-centred approaches to care.

## 5. Conclusions

This study highlights the similarities and differences between ODI and SRS-22 in assessing disability and quality of life in patients with scoliosis and low back pain. While both instruments provide valuable information, their distinct focuses suggest that a combined approach may be optimal for clinical assessment. Gender differences and scoliosis severity influence questionnaire responses, underscoring the need for personalised evaluation strategies. Future research should explore these relationships and refine the PROMs selection to enhance patient-centred care in spinal deformities.

## Figures and Tables

**Table 1:** Descriptive data are presented for the pathology groups and by gender within each specific group. Data are reported as the number of individuals, median (interquartile range), and range.

	Scoliosis + Low Back Pain	Low Back Pain	*p*-Value
**All subjects (N = 1092)**	552	540	-
Gender (females/males)	446/73	356/217	<0.001
Age (years)	64 (16), 50–91	62 (17), 50–93	0.038
BMI (kg/m^2^)	24 (5), 16–43	25 (6), 16–55	<0.001
Cobb angle (°)	39 (31), 0–107	-	-
	Females	Males	*p*-value
**Scoliosis + low back pain**			
Age (years)	63 (14), 50–89	65 (19), 51–91	0.937 (ns)
BMI (kg/m^2^)	23 (5), 16–43	26 (4), 20–41	<0.001
Cobb angle (°)	41 (31), 0–107	28 (28), 8–73	<0.001
**Low back pain**			
Age (years)	63 (16), 50–92	60 (14), 50–93	0.054 (ns)
BMI (kg/m^2^)	25 (6), 16–55	27 (5), 18–41	<0.001

‘ns’, not significant *p*-value.

**Table 2:** ODI results, including the total percentage score and scores for specific items, are presented as median (interquartile range). The dataset includes N = 1092 subjects.

	Scoliosis + Low Back Pain	Low Back Pain	*p*-Value
Total score (%)	24 (22)	24 (20)	0.134 (ns)
Pain	3 (1)	3 (1)	0.357 (ns)
Personal care	1 (1)	1 (2)	0.999 (ns)
Lifting	3 (2)	3 (2)	0.996 (ns)
Walking	2 (2)	2 (2)	0.285 (ns)
Sitting	2 (2)	2 (2)	0.077 (ns)
Standing	2 (2)	2 (1)	0.075 (ns)
Sleeping	2 (1)	2 (0)	<0.001
Social life	2 (2)	2 (2)	0.127 (ns)
Travelling	2 (1)	2 (1)	0.476 (ns)
Employment/Homemaking	3 (1)	3 (1)	0.796 (ns)

‘ns’ indicates a not-significant *p*-value. Differences in total scores were tested using the Wilcoxon rank-sum test, while differences in specific item scores were analysed using Mood’s median test.

**Table 3:** SRS-22 results, including the total score and scores for specific items, are presented as the number of individuals, median (interquartile range). The dataset includes N = 469 subjects.

	Scoliosis + Low Back Pain	Low Back Pain	*p*-Value
Subjects	346	123	-
Total score	61 (21)	55 (22)	0.006
Function/activity (sum score)	15 (10)	15 (5)	0.018
Mental health (sum score)	15 (10)	15 (9)	0.037
Pain (sum score)	15 (5)	15 (5)	<0.001
Self-image (sum score)	10 (5)	10 (5)	0.174 (ns)

‘ns’ indicates a not-significant *p*-value. Differences in total scores were tested using the Wilcoxon rank-sum test, while differences in specific item scores were analysed using Mood’s median test. ‘-’, indicates a test was not applicable.

**Table 4:** COMI Back total score, pain area score, and graphical rating scale (GRS) scores. Data are presented as median (interquartile range). N = 1092 subjects.

	Scoliosis + Low Back Pain	Low Back Pain	*p*-Value
Total score	5 (3)	5 (3)	<0.001
Pain area	1 (1)	1 (1)	-
GRS back	5 (4)	5 (3)	0.402 (ns)
GRS legs	3 (6)	4 (7)	<0.001
	Females	Males	*p*-value
**Scoliosis + low back pain**			
Total score	5 (4)	4 (3)	<0.001
Pain area	1 (1)	1 (0)	-
GRS back	5 (3)	4 (4)	0.001
GRS legs	3 (6)	1 (4)	0.002
**Low back pain**			
Total score	6 (3)	5 (3)	0.002
Pain area	1 (1)	1 (1)	-
GRS back	6 (3)	5 (4)	<0.001
GRS legs	4 (6)	3 (7)	0.032

‘ns’ indicates a not-significant *p*-value. Differences in total scores and in specific item scores were analysed using Mood’s median test. ‘-’ indicates a test was not applicable due to data distribution.

**Table 5:** Spearman’s correlation coefficients aong ODI, SRS-22, and COMI Back total scores. N = 469 subjects.

	Scoliosis + Low Back Pain	Low Back Pain	*p*-Value
ODI and SRS-22	−0.64 *	−0.49 *	0.04
ODI and COMI	0.77 *	0.8 *	0.485 (ns)
SRS-22 and COMI	−0.62 *	−0.53 *	0.215 (ns)
	Females	Males	*p*-value
**Scoliosis + low back pain**			
ODI and SRS-22	−0.64 *	−0.62 *	0.788 (ns)
ODI and COMI	0.77 *	0.73 *	0.649 (ns)
SRS-22 and COMI	−0.63 *	−0.6 *	0.804 (ns)
**Low back pain**			
ODI and SRS-22	−0.53 *	−0.28	0.238 (ns)
ODI and COMI	0.76 *	0.83 *	0.463 (ns)
SRS-22 and COMI	−0.52 *	−0.49 *	0.91 (ns)

*, indicates a coefficient significantly different from zero. The *p*-value for differences in coefficients between the two groups was calculated using Fisher’s z-test. ‘ns’ indicates a non-significant *p*-value.

**Table 6:** Predictive effects of group, gender, age, and BMI on ODI and SRS-22 total scores, evaluated using a generalised linear regression model.

	Group	Gender	Age	BMI
**ODI total score** (N = 1092)				
Estimate *	1.86	6.37	0.29	0.62
*p*-value	0.055 (ns)	<0.001	<0.001	<0.001
Partial correlation with ODI%	0.07	0.21	0.15	0.19
**SRS total score** (N = 469)				
Estimate *	−6.35	−6.79	−0.28	−0.48
*p*-value	<0.001	0.005	0.002	0.023
Partial correlation with ODI%	−0.14	−0.19	−0.17	−0.13

* A positive estimate value for the ‘Group’ variable indicates a greater increasing effect on ODI in the LBP group compared to the SLBP group. A positive estimate value for the ‘Gender’ variable indicates a greater increasing effect in females compared to males. *p*-values for the estimates were tested for significance using a *t*-test. ‘ns’ indicates a non-significant *p*-value. Spearman’s partial correlation coefficients are also reported.

**Table 7:** Predictive effects of gender, age, and BMI on ODI and SRS-22 total scores within the two groups, evaluated using a generalised linear regression model.

		Gender	Age	BMI
**ODI** (N = 1092)	**scoliosis + low back pain** (N = 519)			
	Estimate *	7.81	0.30	0.79
	*p*-value	<0.001	<0.001	<0.001
	Partial correlation with ODI	0.21	0.14	0.22
	**low back pain** (N = 573)			
	Estimate *	5.68	0.29	0.49
	*p*-value	<0.001	<0.001	<0.001
	Partial correlation with ODI	0.22	0.16	0.15
**SRS-22** (N = 469)	**scoliosis + low back pain** (N = 346)			
	Estimate *	−7.19	−0.28	−0.64
	*p*-value	0.007	0.003	0.005
	Partial correlation with SRS-22	−0.17	−0.18	−0.17
	**low back pain** (N = 123)			
	Estimate *	−6.77	−0.30	−0.10
	*p*-value	0.208 (ns)	0.171 (ns)	0.841 (ns)
	Partial correlation with SRS-22	−0.21	−0.21	−0.01

* A positive estimate value for the ‘Gender’ variable indicates a greater increasing effect in females compared to males. *p*-values for the estimates were tested for significance using a *t*-test. ‘ns’ indicates a non-significant *p*-value. Spearman’s partial correlation coefficients are also reported.

## Data Availability

The data presented in this study are openly available in Zenodo at https://doi.org/10.5281/zenodo.17423917.

## Appendix A

**Table A1:** ODI results by gender, including the total percentage score and scores for specific items, are presented as median (interquartile range). The dataset includes N = 1092 subjects.

	Scoliosis + Low Back Pain			Low Back Pain		
	Females	Males	*p*-Value	Females	Males	*p*-Value
Total score (%)	24 (22)	18 (20)	0.003	26 (22)	22 (18)	<0.001
Pain	3 (1)	2 (1)	0.076 (ns)	3 (1)	2 (1)	<0.001
Personal care	1 (2)	1 (1)	-	1 (2)	1 (1)	-
Lifting	3 (2)	2 (2)	0.02	3 (2)	2 (2)	0.002
Walking	2 (2)	1 (1)	0.015	2 (2)	1 (1)	0.013
Sitting	2 (2)	2 (2)	0.126 (ns)	2 (2)	2 (2)	<0.001
Standing	3 (2)	2 (1)	0.054 (ns)	2 (2)	2 (2)	<0.001
Sleeping	2 (1)	2 (1)	0.045	2 (0)	2 (1)	0.008
Social life	2 (2)	2 (2)	0.107 (ns)	3 (2)	2 (2)	0.119 (ns)
Travelling	2 (1)	2 (1)	<0.001	2 (1)	2 (1)	0.251 (ns)
Employment/Homemaking	3 (1)	2 (2)	0.006	3 (1)	2 (1)	<0.001

‘ns’ indicates not significant *p*-value. Differences in total scores were tested using the Wilcoxon rank-sum test, while differences in specific item scores were analysed using Mood’s median test. ‘-’ indicates a test was not applicable due to data distribution.

**Table A2:** Descriptive data and ODI scores for subject groups with and without the SRS-22 questionnaire. Data are reported as the number of individuals, median (interquartile range), and range.

	With SRS-22	Without SRS-22	*p*-Value
Subjects (N = 1092)	469	623	-
Gender (females/males)	408/61	394/229	<0.001
Age (years)	63 (16), 50–92	63 (16), 50–93	0.381 (ns)
BMI (kg/m^2^)	24 (6), 16–41	25 (6), 17–55	<0.001
ODI total score (%)	25 (20), 0–76	24 (20), 0–88	0.573 (ns)

‘ns’, not significant *p*-value.

**Table A3:** SRS-22 results by gender, including the total score and scores for specific items. Data are presented as median (interquartile range). N = 469 subjects.

	Scoliosis + Low Back Pain			Low Back Pain		
	Females	Males	*p*-Value	Females	Males	*p*-Value
Total score	60 (21)	66 (15)	0.014	54 (20)	64 (24)	0.135 (ns)
Function/activity (sum score)	15 (6)	15 (5)	0.332 (ns)	15 (5)	15 (10)	0.136 (ns)
Mental health (sum score)	15 (6)	20 (5)	0.074	15 (5)	15 (5)	0.059 (ns)
Pain (sum score)	15 (5)	15 (5)	0.332 (ns)	15 (5)	15 (5)	0.517 (ns)
Self-image (sum score)	10 (5)	15 (5)	0.846 (ns)	10 (5)	15 (5)	0.637 (ns)

‘ns’ indicates not significant *p*-value. Differences in total scores were tested using the Wilcoxon rank-sum test, while differences in specific item scores were analysed using Mood’s median test.

**Table A4:** Descriptive data within the SLBP group are presented by scoliosis severity and by gender. Data are reported as the number of individuals, median (interquartile range), and range.

	Cobb Angle <30°	Cobb Angle >30°	*p*-Value
All subjects (N = 411)	170	241	-
Gender (females/males)	133/37	214/27	0.004
Age (years)	63 (17), 51–89	63 (12), 50–87	0.825 (ns)
BMI (kg/m^2^)	24 (6), 18–43	23 (5), 16–41	0.034
Cobb angle (°)	19 (10), 10–30	51 (22), 31–107	<0.001
	Females	Males	*p*-value
**Cobb angle <30°**			
Age (years)	63 (16), 51–89	60 (17), 51–85	0.124 (ns)
BMI (kg/m^2^)	24 (6), 18–43	26 (4), 20–33	0.028
Cobb angle (°)	20 (10), 10–30	15 (9), 10–30	<0.001
**Cobb angle >30°**			
Age (years)	63 (12), 50–87	66 (16), 52–84	0.668 (ns)
BMI (kg/m^2^)	23 (4), 16–37	26 (5), 20–41	<0.001
Cobb angle (°)	51 (22), 31–107	44 (16), 31–73	0.016

‘ns’, not significant *p*-value. ‘-’ not applicable comparison.

**Table A5:** Predictive effects of gender, age, and BMI on ODI and SRS-22 total scores within the two scoliosis severity subgroups, evaluated using a generalised linear regression model.

		Gender	Age	BMI
**ODI** (N = 411)	**Cobb angle <30°** (N = 170)			
	Estimate *	6.79	0.4	0.58
	*p*-value	0.014	0.001	0.022
	Partial correlation with ODI	0.22	0.17	0.17
	**Cobb angle >30°** (N = 241)			
	Estimate *	5.74	0.2	0.86
	*p*-value	0.054 (ns)	0.058 (ns)	<0.001
	Partial correlation with ODI	0.15	0.11	0.24
**SRS-22** (N = 268)	**Cobb angle <30°** (N = 75)			
	Estimate *	−6.8	−0.4	−1.02
	*p*-value	0.307 (ns)	0.099 (ns)	0.149 (ns)
	Partial correlation with SRS-22	−0.18	−0.13	−0.15
	**Cobb angle >30°** (N = 193)			
	Estimate *	−11.29	−0.25	−0.71
	*p*-value	<0.001	0.032	0.009
	Partial correlation with SRS-22	−0.25	−0.12	−0.22

* A positive estimate value for the ‘Gender’ variable indicates a greater increasing effect in females compared to males. *p*-values for the estimates were tested for significance using a *t*-test. ‘ns’ indicates a non-significant *p*-value. Spearman’s partial correlation coefficients are also reported.
